# Lipoprotein(a) Is Associated with Thrombus Burden in Culprit Arteries of Younger Patients with ST-Segment Elevation Myocardial Infarction

**DOI:** 10.1159/000529600

**Published:** 2023-02-09

**Authors:** Dipen M. Sankhesara, Nick S.R. Lan, Poppy Gilfillan, Elly Zounis, Saumya Rajgopal, Dick C. Chan, Jing Pang, Graham S. Hillis, Gerald F. Watts, Carl J. Schultz

**Affiliations:** ^a^Department of Cardiology, Royal Perth Hospital, Perth, Washington, Australia; ^b^Medical School, University of Western Australia, Perth, Washington, Australia

**Keywords:** Lipoprotein(a), Myocardial infarction, Lipids, Coronary artery disease, Thrombosis

## Abstract

**Background:**

Lipoprotein(a) (Lp[a]) is a risk factor for cardiovascular disease. The burden of thrombus in ST-segment elevation myocardial infarction (STEMI) has implications on treatment and outcomes. However, the association between Lp(a) and atherothrombosis in STEMI remains unclear.

**Objectives:**

The aim of the study was to determine the association between Lp(a) and culprit artery thrombus burden in younger patients with STEMI.

**Methods:**

This was a single-center study of 83 patients aged <65 years with STEMI between 2016–2018 who underwent percutaneous coronary intervention and measurement of Lp(a); those receiving thrombolytic therapy were excluded. Thrombus burden in the culprit artery was determined angiographically using the Thrombolysis In Myocardial Infarction score and classified as absent-to-small, moderate, or large. Elevated Lp(a) was defined as plasma mass concentration >30 mg/dL. Multivariate analysis was performed adjusting for cardiovascular risk factors.

**Results:**

The mean age was 48.0 ± 8.4 years, and 78.3% were male. Thirteen (16%), 9 (11%), and 61 (73%) patients had small, moderate, or large thrombus burden, respectively, and 34 (41%) had elevated Lp(a). Elevated Lp(a) was associated with greater thrombus burden compared to normal Lp(a) (large burden 85% vs. 65%; *p* = 0.024). Elevated Lp(a) was associated with moderate or large thrombus in univariate (OR 10.70 [95% CI 1.32–86.82]; *p* = 0.026) and multivariate analysis (OR 10.33 [95% CI 1.19–89.52]; *p* = 0.034). Lp(a) was not associated with culprit artery or stenosis location according to culprit artery.

**Conclusions:**

Elevated Lp(a) is associated with greater thrombus burden in younger patients with STEMI. The finding of this observational study accords with the thrombotic and anti-fibrinolytic properties of Lp(a). A causal relationship requires verification.

## Introduction

Lipoprotein(a) (Lp[a]) is a genetically determined low-density lipoprotein (LDL)-like particle bound covalently to apolipoprotein(a) (apo[a]) [1]. Mendelian randomization studies demonstrate that Lp(a) is a causal risk factor for atherosclerotic cardiovascular disease [2]. Imaging studies have shown that Lp(a) is associated with increased burden and progression of coronary artery plaque, considered to be mediated by its LDL-like moiety [3]. In addition to its pro-atherosclerotic effects, Lp(a) has been considered a pro-thrombotic and anti-fibrinolytic molecule, mediated by the apo(a) moiety, supported by its homology with plasminogen [4]. While high Lp(a) is not a risk factor for venous thromboembolism [1], the association of Lp(a) with acute arterial thrombosis remains unclear.

At present, there is limited literature evaluating Lp(a) levels and its association with coronary artery thrombosis in vivo [4]. In the setting of acute ST-segment elevation myocardial infarction (STEMI), plaque rupture typically causes localized, often occlusive thrombus formation. The burden of thrombus at time of STEMI can be determined visually at the time of angiography and has important clinical implications with regard to complications of percutaneous coronary intervention (PCI) such as distal embolization, potential consideration of thrombectomy, and long-term outcomes [5]. We hypothesized that Lp(a) levels are associated with greater thrombus burden at coronary angiography in patients with STEMI.

## Materials and Methods

Patients who presented with STEMI between January 2016 and December 2018, who underwent primary PCI, were aged <65 years, and who had a Lp(a) measurement performed during the index admission were retrospectively identified by case record review at Royal Perth Hospital, Australia. Approval was granted by the Local Institutional Review Board. This age group was chosen to study the effects of Lp(a) in patients with premature coronary artery disease. In the year 2016, Lp(a) was measured in consecutive patients over a 6-month period for a separate study. Lp(a) measurement was performed using an automated latex-enhanced immunoassay (Quantia Lp[a]) on an Abbott Architect c16000 Analyzer (Abbott Diagnostics, Abbott Park, IL, USA). This assay employs a rabbit polyclonal antibody and is sensitive to variation in apo(a) isoform size.

PCI for STEMI is performed by certified cardiologists with training in intervention, and coronary angiography is performed using standard techniques and angulated views. Images were reviewed retrospectively by a cardiologist blinded to Lp(a) results. The burden of thrombus in the culprit coronary artery was determined on angiographic images performed prior to PCI, using the validated Thrombolysis In Myocardial Infarction (TIMI) thrombus grade, and classified as absent to small, moderate, or large depending on the angiographically apparent luminal filling defect [5]. A prior study showed that patients with moderate or large thrombus in the culprit artery at time of STEMI might derive benefit from rheolytic thrombectomy before stenting compared with direct stenting alone [5]. Although thrombectomy is not routinely performed, moderate or large thrombus burden was chosen as the outcome given its potential consequences for future management. The location of the culprit stenosis was defined as being in the proximal, mid, or distal segment of the coronary artery. All other data were collected from review of medical records. Patients undergoing primary PCI at our institution are routinely given dual anti-platelet therapy (aspirin with either clopidogrel or ticagrelor) and intravenous unfractionated heparin loading prior to angiography as per local STEMI protocol. Patients given thrombolytic therapy were excluded.

Statistical analyses were performed using SPSS (IBM Corp. SPSS version 21, Armonk, NY, USA). Descriptive data are presented as number (%) or mean ± standard deviation, as the Kolmogorov-Smirnov test was not significant for the continuous variables. Differences between groups were analyzed using Student's *t* test, Pearson's χ^2^ test, or Fisher's exact test. An elevated Lp(a) was prospectively defined as a plasma mass concentration >30 mg/dL, as this level has been shown to be associated with increased risk of myocardial infarction [6]. As Lp(a) exhibits a positively skewed distribution, Lp(a) was natural log-transformed for analysis as a continuous variable. Multivariate logistic regression was used to assess whether log-transformed Lp(a) (continuous variable) or elevated Lp(a) (categorical variable) was independently associated with moderate or large thrombus burden, with data presented as odds ratios (OR) and 95% confidence intervals (CI). Statistical significance was defined as a two-sided *p* value <0.05.

## Results

Eighty-three patients with mean age 48.0 ± 8.4 years and *n* = 65 (78%) male were included. Elevated Lp(a) was detected in 34 (41%). Baseline characteristics stratified according to Lp(a) status are presented in Table 1, with no significant differences in cardiovascular risk factors including age, sex, hypertension, diabetes mellitus, current smoking, LDL cholesterol, prior myocardial infarction, and anti-thrombotic therapy prior to presentation.

Overall, 13 (16%), 9 (11%), and 61 (73%) patients had small, moderate, and large burden of thrombus in the culprit artery, respectively. Patients with elevated Lp(a) were more likely to have greater severity of thrombus burden (*p* = 0.024), where large thrombus burden was seen in 85% with versus 65% of those without elevated Lp(a). Log-transformed Lp(a) was associated with an increased risk of moderate or large thrombus burden in univariate (OR 11.25 [95% CI 1.52–83.17]; *p* = 0.018) and multivariate analysis (Table 2, model 1: OR 2.60 [95% CI 1.06–6.35]; *p* = 0.037). Elevated Lp(a) was associated with increased risk of moderate or large thrombus burden in univariate analysis (OR 10.70 [95% CI 1.32–86.82]; *p* = 0.026) and multivariate analysis (Table 2, model 2: OR 10.33 [95% CI 1.19–89.52]; *p* = 0.034). There were no significant associations between elevated Lp(a) and left ventricular ejection fraction, culprit artery, or location of stenosis according to coronary artery segment.

## Discussion

This study is the first to report that elevated levels of Lp(a) are associated with a greater thrombus burden in the culprit coronary artery in patients presenting with STEMI. This supports the hypothesis that arterial thrombus formation following plaque rupture in STEMI may be influenced by Lp(a).

A previous study reported that higher Lp(a) levels were associated with lower coronary artery patency rates in patients with STEMI not treated with reperfusion therapy, while another study found an association with lower spontaneous recanalization of the infarct artery on diagnostic angiography prior to primary PCI in patients with STEMI [7, 8]. Interestingly, Lp(a) levels did not affect the success of thrombolytic therapy administered during acute myocardial infarction in small studies [9]. These studies also had relatively young populations (mean age 50–60 years), and thus, the data suggest that Lp(a) may impair endogenous fibrinolysis or may be pro-thrombotic in patients with STEMI [7, 8, 9]. The apo(a) moiety of Lp(a) promotes prothrombin activation and platelet aggregation, factors which are important mediators of thrombus formation following plaque rupture, which is likely of relevance to the present study [4]. The mechanisms by which Lp(a) promotes atherothrombosis are complex and have been reviewed elsewhere [4].

The current findings may have potential clinical implications with respect to the intensity and duration of anti-platelet or anti-thrombotic therapies for patients with STEMI and elevated Lp(a). A pro-thrombotic state contributes to residual cardiovascular risk. In a large retrospective registry, patients with stable coronary artery disease and Lp(a) >30 mg/dL were found to have reduced cardiovascular events with prolonged duration (>1 year) of dual anti-platelet therapy after PCI [10]. Clinically relevant bleeding was not increased in those with elevated Lp(a) with prolonged dual anti-platelet therapy, in contrast to those with normal Lp(a) [10]. While addition of low-dose rivaroxaban to aspirin for secondary prevention significantly reduced cardiovascular events in a large randomized trial, Lp(a) was not measured [11]. Whether secondary prevention patients with elevated Lp(a) derive greater benefits from prolonged or intensified anti-platelet or anti-thrombotic therapies remains unclear. Likewise, in a primary prevention setting, the potential for aspirin to prevent cardiovascular events in patients with Lp(a)-associated genetic variants that elevate Lp(a) is another area worthy of additional research [12]. Lp(a)-lowering therapies targeting hepatic synthesis of apo(a) at an RNA level (i.e., pelacarsen and olpasiran) may also impact thrombotic risk after an acute coronary syndrome, with outcome trials currently ongoing in patients with cardiovascular disease (NCT04023552 and NCT05581303).

The results of the current study provide further support for the routine measurement of Lp(a) in younger patients with STEMI. An Lp(a) level of 30 mg/dL is approximately the 75th percentile in white populations, but elevated Lp(a) (defined as >30 mg/dL) was detected in 41% of patients in this Australian study [1]. This suggests that the yield of detecting elevated Lp(a) is higher in younger patients with STEMI compared to the general population. In secondary prevention, elevated Lp(a) level is a risk factor for cardiovascular events, even among patients treated with statins [1]. Thus, patients with cardiovascular disease and elevated Lp(a) require more intensive risk factor management. Current guidelines also recommend that Lp(a) be measured in people with premature cardiovascular disease or at least once in an adult person's lifetime [1]. However, whether the inclusion of Lp(a) improves the performance of risk prediction equations after STEMI requires further research [1].

Limitations of this study include its small sample size and retrospective analysis at a single center. In addition, Lp(a) was not measured routinely in all patients with STEMI during the study period; thus, this cohort may be affected by selection bias. Lp(a) was measured at a single time point, as Lp(a) concentration is considered relatively stable throughout one's lifetime [1]. However, during acute myocardial infarction, Lp(a) levels may vary due to the acute phase reaction [13]. Furthermore, Lp(a) was measured using an assay that is affected by Lp(a) isoform size. Guidelines recommend measurement using an immunochemical assay where the effect of apo(a) isoform size is minimized [1]. However, we previously reported a significant correlation between the Quantia assay and a liquid chromatography-mass spectrometry method [14]. Reliable data on time from symptoms to PCI or infarct size, apart from left ventricular function on echocardiography, were not available. Although very low Lp(a) levels are associated with increased risk of diabetes mellitus, a statistically significant difference was not observed potentially due to small sample size [1]. Other lipid biomarkers such as apolipoprotein A1, apolipoprotein B, and paraoxonase-1 were not available, but have recently been shown to be associated with susceptibility to STEMI in young people [15]. Lastly, other mediators of thrombosis, fibrinolysis, and inflammation which may add to risk prediction, such as fibrinogen, D-dimer, homocysteine, C-reactive protein, oxidized phospholipids, and thrombophilia screen were not available, as these are not routinely measured.

In conclusion, an elevated Lp(a) level is associated with greater thrombus burden in culprit arteries in younger patients presenting with STEMI. The finding of this observational study concurs with the pro-thrombotic and anti-fibrinolytic properties of Lp(a) and may have important clinical implications if a causal relationship is verified.

## Statement of Ethics

The study complies with the Declaration of Helsinki and has been approved by the Royal Perth Hospital Institutional Review Board (GEKO 40393). The need for informed consent was waived due to retrospective analysis of de-identified data.

## Conflict of Interest Statement

NSRL has received research funding from Sanofi as part of a Clinical Fellowship in Endocrinology and Diabetes, education support from Amgen, Bayer, Boehringer Ingelheim, and Eli Lilly, and speaker honoraria from Sanofi, Boehringer Ingelheim, and Eli Lilly. GFW has received honoraria for advisory boards and research grants from Amgen, Arrowhead, Esperion, Novartis, Pfizer, Sanofi, and Regeneron.

## Funding Sources

This study was not funded.

## Author Contributions

Conceptualization and design, and methodology and project administration: D.M.S. and C.J.S. Data curation: D.M.S., P.G., E.Z., and S.R. Formal analysis: N.S.R.L. and D.C.C. resources: G.S.H., D.C.C., J.P., G.F.W., and C.J.S. Interpretation of data: D.M.S., N.S.R.L., D.C.C., J.P., G.S.H., G.F.W., and C.J.S. Writing − original draft: D.M.S. and N.S.R.L. Writing − review and editing: All authors.

## Data Availability Statement

Data are not publicly available due to ethical reasons. Further inquiries can be directed to the corresponding author.

## Figures and Tables

**Table 1:** Characteristics of the study cohort

Characteristic	Overall (*n* = 83)	Lp(a) >30 mg/dL (*n* = 34)	Lp(a) ≤30 mg/dL (*n* = 49)	*p* value
Age	48.0±8.4	48.8±8.3	47.5±8.6	0.506
Male sex, *n* (%)	65 (78.3)	23 (67.6)	42 (85.7)	0.050
Hypertension, *n* (%)	37 (44.6)	14 (41.2)	23 (46.9)	0.603
Diabetes mellitus, *n* (%)	26 (31.3)	9 (26.5)	17 (34.7)	0.427
Current smoker, *n* (%)	42 (50.6)	15 (44.1)	27 (55.1)	0.325
Previous MI, *n* (%)	5 (6.0)	4 (11.8)	1 (2.0)	0.154
Anti-platelet therapy prior*, *n* (%)	7 (8.4)	4 (11.8)	4 (8.2)	0.711
LDL cholesterol	3.6±1.0	3.7±1.1	3.6±1.0	0.675
Creatinine, µmol/L	78.8±17.6	78.7±17.7	79.0±17.7	0.951
Hemoglobin, g/L	144.9±20.1	141.6±26.6	147.2±13.7	0.260
TIMI thrombus burden, *n* (%)				
Small	13 (15.7)	1 (2.9)	12 (24.5)	0.024
Moderate	9 (10.8)	4 (11.8)	5 (10.2)	
Large	61 (73.5)	29 (85.3)	32 (65.3)	
Culprit artery, *n* (%)				
LAD**	39 (47.0)	15 (44.1)	24 (49.0)	0.560
Circumflex	15 (18.1)	8 (23.5)	7 (14.3)	
RCA	29 (34.9)	11 (32.4)	18 (36.7)	
Culprit location, *n* (%)				
Proximal	38 (45.8)	16 (47.1)	22 (44.9)	0.821
Mid	25 (30.1)	15 (44.1)	20 (40.8)	
Distal	10 (12.0)	3 (8.8)	7 (14.3)	
LVEF (%)	48.1±10.2	50.2±9.7	46.7±10.4	0.140

LAD, left anterior descending artery; LDL, low-density lipoprotein; LVEF, left ventricular ejection fraction; MI, myocardial infarction; RCA, right coronary artery; TIMI, thrombolysis in myocardial infarction.

* No patients were receiving anticoagulant therapy prior to admission.

** Includes 1 patient with left main stenosis.

**Table 2:** Multivariate logistic regression analysis of Lp(a) as a predictor of moderate to large thrombus burden

Variable	OR (95% CI)	*p* value
Model 1		
Log [Lp(a)]	2.60 (1.06–6.35)	0.037
Age	0.96 (0.88–1.04)	0.301
Male sex	0.30 (0.03–2.89)	0.299
Hypertension	2.28 (0.54–9.64)	0.261
Diabetes mellitus	0.34 (0.08–1.47)	0.149
Current smoker	1.35 (0.36–5.07)	0.657
LDL cholesterol	1.09 (0.58–2.05)	0.786
Model 2		
Lp(a) >30 mg/dL	10.33 (1.19–89.52)	0.034
Age	0.95 (0.87–1.03)	0.204
Male sex	0.29 (0.03–2.79)	0.284
Hypertension	2.46 (0.56–10.79)	0.233
Diabetes mellitus	0.31 (0.07–1.37)	0.122
Current smoker	1.57 (0.41–6.03)	0.515
LDL cholesterol	1.05 (0.56–1.96)	0.873

Lp(a), lipoprotein(a); LDL, low-density lipoprotein.
